# Virtual adaptation of traditional healthcare quality improvement training in response to COVID-19: a rapid narrative review

**DOI:** 10.1186/s12960-020-00527-2

**Published:** 2020-10-28

**Authors:** Zuneera Khurshid, Aoife De Brún, Gemma Moore, Eilish McAuliffe

**Affiliations:** 1 UCD Centre for Interdisciplinary Research, Education, and Innovation in Health Systems (UCD IRIS), School of Nursing, Midwifery and Health Systems, University College Dublin, Room B111, Dublin, Ireland; 2 UCD Centre for Interdisciplinary Research, Education, and Innovation in Health Systems (UCD IRIS), School of Nursing, Midwifery and Health Systems, University College Dublin, Dublin, Ireland; 3 National Quality Improvement Team, Evidence for Improvement, Health Service Executive, Stewarts Hospital, Mill Lane, Palmerstown, Dublin, D20HY57 Ireland

**Keywords:** Quality improvement, Online learning, Medical education, Quality improvement training

## Abstract

**Background:**

Information and communication technology are playing a major role in ensuring continuity of healthcare services during the COVID-19 pandemic. The pandemic has also disrupted healthcare quality improvement (QI) training and education for healthcare professionals and there is a need to rethink the way QI training and education is delivered. The purpose of this rapid evidence review is to quickly, but comprehensively collate studies to identify what works and what does not in delivering QI training and education using distance learning modalities.

**Methods:**

Three healthcare databases were searched along with grey literature sources for studies published between 2015 and 2020. Studies with QI training programmes or courses targeting healthcare professionals and students with at least one component of the programme being delivered online were included.

**Results:**

A total of 19 studies were included in the review. Most studies had a mixed methods design and used blended learning methods, combining online and in-person delivery modes. Most of the included studies reported achieving desired outcomes, including improved QI knowledge, skills and attitudes of participants and improved clinical outcomes for patients. Some benefits of online QI training delivery include fewer required resources, reduced need for on-site instructors, increased programme reach, and more control and flexibility over learning time for participants. Some limitations of online delivery modes include limited learning and networking opportunities, functional and technical problems and long lead time for content adaptation and customisation.

**Discussion:**

The review highlights that distance learning approaches to QI help in overcoming barriers to traditional QI training. Some important considerations for those looking to adapt traditional programmes to virtual environments include balancing virtual and non-virtual methods, using suitable technological solutions, customising coaching support, and using multiple criteria for programme evaluation.

**Conclusion:**

Virtual QI and training of healthcare professionals and students is a viable, efficient, and effective alternative to traditional QI education that will play a vital role in building their competence and confidence to improve the healthcare system in post-COVID environment.

## Background

The COVID-19 pandemic is rapidly transforming the landscape of the healthcare system and virtual healthcare solutions are playing a key role in this change [1]. It has also presented unique challenges in the healthcare quality improvement (QI) sphere and highlighted the need for a dynamic approach that enables QI structures and policies to adapt to the pandemic environment [2]. QI principles offer useful strategies for implementing and sustaining meaningful change [3] and staff trained in QI principles can play a critical role in responding to these emerging challenges by accelerating the pace of learning [4]. QI is increasingly being recognised as an important skill for healthcare professionals [5] and is an important component of medical education and training [6]. The immediate focus of the COVID-19 response of the healthcare sector has been on ensuring continuity of care for patients and communities. However, the pandemic has also had a profound impact on medical education delivery and how healthcare professionals will be educated in future [7]. As researchers and practitioners rush to explore ways to support healthcare professionals during the pandemic, it is also important to rethink the way QI training and education is delivered to healthcare professionals.

Rather than viewing COVID-19 as a disruption to healthcare QI education and training, it can be considered as an opportunity to improve distance learning techniques and benefit from digital hyper-connectivity to enhance education delivery that can extend into the post-pandemic environment [8]. Application of technology-enhanced learning is often cited as a pedagogical advancement for a curricular transformation of medical education [9]. However, the pandemic has made it an inescapable necessity for the healthcare system to adapt to virtual ways of working.

Online learning platforms have the potential to bring healthcare professionals together to share knowledge and collaborate in QI teaching, learning and education [10]. Beyond the pandemic situation, well designed, self-directed e-learning programmes which are responsive to the dynamic healthcare sector, may lead to better knowledge retention as compared to traditional didactic lectures [11]. There is much to be understood about the usefulness of distance learning modalities in effectively delivering QI training. The purpose of this rapid narrative review is to collate studies to identify what works and what does not in delivering QI training and education using distance learning modalities. A rapid evidence assessment summarises research findings in a systematic manner, within time and resource constraints and is suited to the current situation [12].

The review aims to answer the following questions:

- What distance learning modalities are being used to train healthcare staff and students in QI methods?
- What is the efficacy of distance learning QI programmes?
- What were the advantages and limitations in delivering QI programmes using distance learning modalities?

By answering these questions, we aim to synthesise guidelines and recommendations for those who are dealing with the challenge of adapting and delivering QI training to healthcare professionals through distance learning modes.

## Methods

**Fig. 1:**
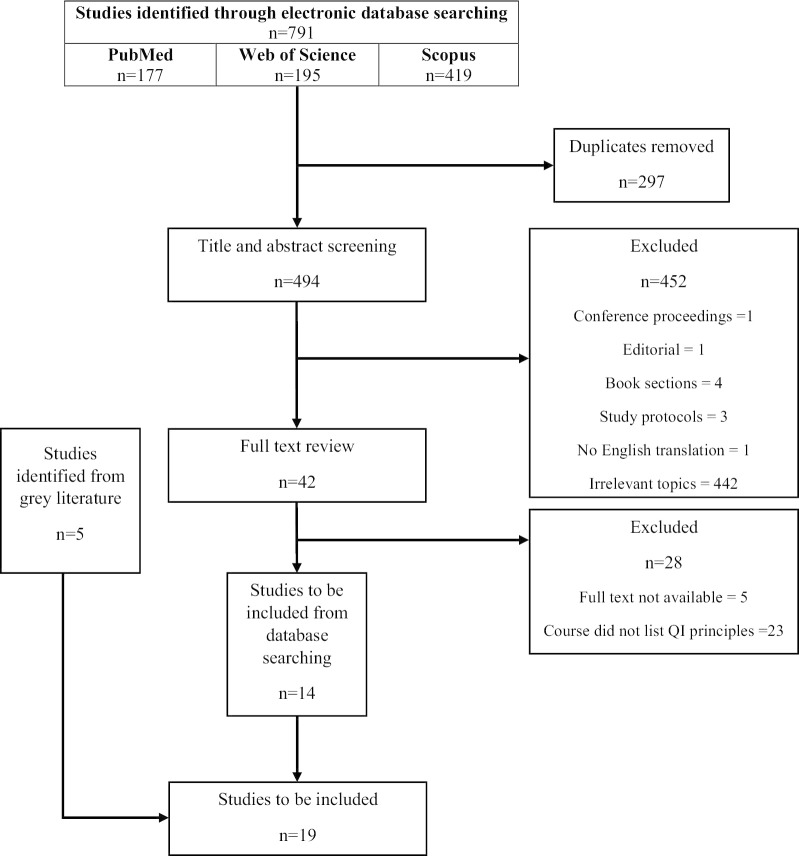
PRISMA diagram. Study screening and selection process

Three databases (PubMed, Web of Science and Scopus) were searched to identify studies published between 2015 and 2020. The inclusion criteria were QI training programmes or courses targeting healthcare professionals and students with at least one component of the programme being delivered online. Only studies with primary data were included. Conference proceedings, editorials, protocols, and book sections were excluded. Studies that did not explicitly teach QI principles in the programme were excluded. Papers with no full text available or no English translation available were also excluded. Reference lists of included papers and grey literature search of Google Scholar was also conducted to identify further papers. The search and screening processes are documented in Fig. 1 (Prisma Diagram) and search strategy is presented in Additional file 1: Search strategy. The critical appraisal of the studies was conducted using the Quality Improvement Minimum Quality Criteria Set (QI-MQCS)—Version 1.0 tool which possesses acceptable psychometric properties for critical appraisal to support systematic reviews containing diverse quality improvement intervention (QII) evaluations [13].

## Results

The summary of the 19 studies included in this evidence synthesis is presented in Table 1.

**Table 1:** Summary of studies

Study characteristics			Population description		Intervention description			Outcomes	
Study ID	Location	Study design	Population	Sample size	Training purpose	Intervention type	Delivery modes	Evaluation of outcomes	Outcomes achieved
Baernholdt-2017 [14]	United States	Mixed methods	Interprofessional health care teams	40	Training interprofessional health care teams to lead QI projects using PDSA methodology	Interprofessional Quality Improvement Training Program	Seminars, online modules, bimonthly meetings, QI project work	Participation Learner reactions to training Participants’ QI knowledge, attitudes, behaviours Patient safety outcomes	19 out of 22 teams completed the programme Higher QI self-efficacy post-programme Program and sessions rated favourably Improvements in clinical settings
Baxley-2016 [15]	United States	Mixed methods	Interprofessional group of faculty	27	Preparing faculty to lead frontline clinical transformation	Teachers of Quality Academy Professional development program	Online, didactic, small-group, experiential learning, QI project, QI symposium	Progress of QI initiatives Incorporation of educational modules into curriculum Production of scholarly products by participants Participants’ QI knowledge, attitudes, behaviours Patient safety outcomes Interprofessional practice	All participants completed QI projects 70% participants engaged in design and delivery of curriculum Participants applied new knowledge and skills in educational initiatives development
Bonnes-2017 [16]	United States	Prospective validation study	Internal medicine residents	143	Educating trainees on how to successfully improve health care quality	Flipped QI curriculum	Online modules, facilitated small group discussions	Preferences for mode of delivery Past experiences with delivery mode Completion of online modules Participants’ QI knowledge, attitudes, behaviours	Improved perception of FC Participants of FC demonstrated improved QI knowledge compared to the control group FC associated with greater engagement in online modules
Gregory-2018 [17]	United States	Quantitative descriptive	Postdoctoral nurses, post-residency physicians, clinical psychologist	54	Training health care professionals to become leaders in QI	Veterans affairs quality scholars curriculum	Web-based curriculum delivered in real time	Participants’ QI knowledge, attitudes, behaviours Transfer of training Learner reactions to training	Learners satisfied with training Improvements in QI knowledge, attitudes, behaviours Significant improvement in affective transfer but no significant change in cognitive or skill-based transfer
Hafford-Letchfield-2018 [18]	United Kingdom	Mixed methods	Social workers, midwives, community nurses, occupational therapists, dieticians, general and mental health nurses	62	Using digital storytelling method to encourage collaboration for identifying and developing plans for service improvements	Service development and quality improvement module	Digital storytelling pedagogy with online activities and half-day workshops taught face-to-face	Developing digital story Developing action plan to address selected issue Writing improvement plan Experience with delivery mode Patient safety outcomes	Levelling effect in interprofessional collaboration Programme content should focus on communicating service user/patient needs Virtual learning pedagogies encourage co-construction of shared solutions across disciplines Nearly all students created innovative and informative digital stories with genuine practical utility
Hargreaves-2017 [19]	United States	Mixed methods	Primary care, public health, and community leaders and project managers, faculty, project staff	11 teams	Sharing and spreading, evidence-based QI practices to prevent and treat obesity	National Initiative for Children’s Healthcare Quality (NICHQ) Healthy Weight Collaborative	In-person networking events and virtual learning sessions, webinars, coaching calls, peer networking calls, technical assistance calls	Implementation of activities Developing action plans Engagement of community teams Project results Patient outcomes Online module usage patterns Documents submitted by teams	Developed collaborative capacity among teams 34% of Phase 2 teams had an “above average” level of engagement Use of QI methods and performance measures helped teams make progress All teams adopted a healthy weight message, 59% implemented community-wide assessments and plans
Jamal-2017 [20]	United States	Quantitative descriptive	Otolaryngology residents	11	Integrating patient safety and quality improvement into resident education	Patient Safety and Quality Improvement (PSQI) curriculum	Interactive online modules, classroom group discussions, lectures by PSQI experts, self-directed workshops to develop projects	Online module content and quality Number of projects developed Confidence in using QI	IHI online modules are appropriate for patient safety and QI beginners and well accepted by participants Over half of residents found these modules to be ‘‘extremely’’ or ‘‘very’’ worthwhile
Keefer-2016 [21]	United States	Quantitative descriptive	House officers	80	Training house staff about basic QI techniques	Flipped classroom quality improvement curriculum	Online modules and in-person workshops	Participants’ QI knowledge, attitudes, behaviours	Improved QI content knowledge Improved perceived readiness to participate in QI projects
Kennedy-2017 [22]	United States	Mixed methods	Faculty, staff, administrators, supervisors, data managers	60	Undertaking and sharing Continuous Quality improvement techniques	Online quality improvement Information exchange	Web-based portal/website	Experience with delivery mode QI delivery mode effectiveness, efficiency, satisfaction	Results were overall positive and desirable Majority reviewers reported they would use the learning materials, complete quality improvement projects and reported the site would help address quality improvement challenges
Maxwell-2016 [23]	United States	Pretest/posttest control group design	Baccalaureate nursing students	64	Improving knowledge, skills, and attitudes regarding QI and safety	QSEN competencies	Online modules, flipped classroom	Participants’ QI knowledge, attitudes, behaviours, and comfort Safety knowledge, comfort, and attitude	Statistically significant effect between the groups for QI Experimental group had slightly higher knowledge scores than the control group for safety and QI Use of online modules in conjunction with the flipped classroom had a greater effect on increasing QI knowledge than the use of online modules only
Potts-2016 [24]	United States	Mixed methods	Family Medicine residents	23	Integrating residents to actively participate in quality improvement and patient safety activities	Integrated quality improvement residency curriculum	Web-based tutorials, quality improvement projects, small-group sessions	Quality improvement skills Patient safety skills Chronic care management	Participants of full curriculum reported higher use of knowledge Chronic care management and patient safety skill significantly improved for majority items Only one item (designing prospective chart reviews) was significantly improved for the QI skills category
Ramar-2015 [25]	United States	Quantitative	Fellowship trainees	7	Incorporating a QI curriculum into a training program	Flipped classroom (FC) model	Video lessons, half-day session, case examples, a hands-on workshop	Learner reactions to training Participants’ QI knowledge, attitudes, behaviours	Significant improvement in post-FC QI knowledge Overall positive reaction towards FC model
Scales-2016 [26]	United States	Randomised control trial	Resident physicians	422	Increasing learner participation in quality improvement education	QI curriculum	Spaced delivery of interactive healthcare quality questions via email	Participation Participant engagement	Residents in the intervention arm demonstrated greater participation than the control group Percentage of questions attempted at least once was greater in the intervention group versus control group Response time was faster in intervention group Team competition increases resident participation in an online course delivering QI content
Shaikh-2017 [27]	United States	Quantitative descriptive	Residents and faculty	500	Increasing resident and faculty knowledge in QI, patient safety, and care transitions	University of California Health’s Enhancing Quality in Practice online course	Three modules, questions sent on smartphones using an app, or on computers using e-mail	Course completion QI knowledge Patient safety outcomes Preferences for mode of delivery	Learners rated quiz-based system as an effective teaching modality and preferred it to classroom-based lectures Course completion rate between 66–86% Knowledge acquisition scores for, QI, patient safety and care transitions increased after course completion Course best utilised to supplement classroom and experiential curricula
Shelgikar-2017 [28]	United States	Mixed methods	Sleep medicine fellows	7	Developing skills to systematically analyse practice using quality improvement methods, and implement changes	QI curriculum using a flipped classroom	Online modules and group sessions	QI knowledge Confidence in QI application Participation Project completion	All participants completed the curriculum Knowledge of QI concepts and confidence in performing QI activities increased QI projects improved timeliness and quality of care for patients
Sorita-2015 [29]	Canada	Mixed methods	Secretaries, clinical assistants, registered nurses, nurse practitioners, physician assistants, physicians	Not stated	Running Plan-Do-Study-Act cycles to streamline examination process	QI curriculum	Didactics, workshop, online modules, and experiential learning	Improvement in care process	Residents successfully applied QI methods to improve the efficiency of the DOT examination process Total visit time successfully reduced Accuracy of certificate issuance, as proxy for examination quality improved after intervention
Tappen-2018 [30]	United States	Randomised, controlled trial	Nursing Facility Residents	264	Improving the identification, evaluation, and management of acute changes	INTERACT quality Improvement Program	INTERACT tools, online training programme, webinars, an intensive initial training programme, monthly follow-up webinars	Patient safety outcomes	No adverse effects on resident safety No significant differences in safety indicators between intervention and comparison group Intervention NFs with high levels of INTERACT tool use reported significantly lower rates of severe pain
Tartaglia-2015 [31]	United States	Observational study with control group	Fourth-year medical students	34	Improving QI knowledge	QI curriculum	Online modules, reflective writing, discussion with content expert, mentored QI project	Comfort with QI principles Participants’ QI knowledge, attitudes, behaviours Projects completion	Students in the intervention group reported more comfort with their skills in QI Curriculum strength included effective use of classroom time, faculty mentorship, reliance on pre-existing online modules Curriculum is expandable to larger groups and transferable to other institutions
Zubkoff-2019 [32]	United States	Mixed methods	Team leader, senior level support person, nurse, physician, nurse practitioner champion, pharmacist, and physical therapist	60	Enhancing knowledge, infrastructure, and capacity for QI	Virtual breakthrough series collaborative	Webinar-based educational format, open discussion sessions, “Meet and Greet” call with coaches, pre-work calls	Learner reactions to training Report submission Patient safety outcomes	No statistically significant decrease in total fall rates or major injury rates Significant improvement in fall related injury rate Majority were satisfied with the educational calls Minor injury rate decreased significantly Monthly report submission between 65 to 85%

A summary of study characteristics, intervention descriptions, and outcomes of included studies

### Study characteristics

Although, the overarching aim of the included studies was to improve QI skills of healthcare professionals and students, the studies differed in design, evaluation, and analytical methods used. Most studies had a mixed methods design and 17 of the 19 studies were based in the United States. Design of the interventions was also variable; most studies used a blended learning method combining online and in-person modes while only six studies [17, 22, 26, 27, 30, 32] were entirely delivered online. Some blended learning modules conducted classroom-based sessions followed by support through online modules and QI project completion, while others used a flipped curriculum approach where participants completed online modules prior to the in-person sessions such as seminars, workshops, lectures and QI project completion.

### Quality assessment

All included studies were deemed to be of good quality even though some studies did not report on all areas evaluated by the QI-MQCS tool. All studies discussed the rationale behind the intervention, organisational motivation, description of the intervention and implementation approach. Some studies included limited information about describing sustainability or the potential for sustainability of the interventions and explicitly naming the study design. None of the studies were excluded based on quality assessment and a detailed quality assessment is attached in Additional file 2: Quality assessement of included studies.

### Distance learning modes

The online delivery modes used by studies included online modules [14–16, 20–24, 28–31], access to web-based curricula [17], virtual learning environments [18], webinars [19, 30, 32], calls [19, 32], web-based QI portals [22], smartphone apps [27], emails [26, 27], access to package of tools [30], virtual whiteboard [21] and video lessons [25]. Instead of developing their own distance learning content, most studies relied on the completion of the Institute of Healthcare Improvement’s (IHI) online modules [15, 16, 20, 23, 31, 32], many of which are free to use. The rationale behind using IHI’s modules is that it provides a standardised methodology which does not require prior faculty proficiency or entail an increase in educational time commitment [20]. The IHI methodology is designed to help organisations in identifying and closing gaps via a standard improvement methodology [32]. Another advantage is that an institutional subscription to the IHI programme provides access to comprehensive QI training and allows tracking the progress of participants [24]. Some studies adapted IHI modules [29, 32] to their local context while other used self-developed content [17–19, 22, 27, 28, 30]. The major online modalities used are summarised in Table 2.

**Table 2:** Description of online modes

Modality	Description
Flipped curriculum/flipped classroom	Instructional content delivered through online modes before class and class time used for knowledge application [16, 21, 23, 28]
Virtual breakthrough series collaborative	Virtual adaptation of the Institute for Healthcare Improvement (IHI) face-to-face collaborative model through webinar-based educational delivery [32]
Dedicated web portal/QI site	In-house QI sites developed to provide access to QI tools, resources, and training [19, 22]
Interactive online delivery	Didactic lectures delivered live online allowing participants to participate in real time [17, 20]
Video lectures	Pre-recorded didactic lectures made available to participants [25]
Phone/app/email-based methods	QI questions sent out to participants through text messages, phone apps or email [26, 27]
Online modules to supplement classroom delivery	IHI QI modules [14, 15, 24, 31] Self-developed QI modules [19, 29]

Summary of major online modes used by studies in delivering QI training and education

Only a few studies discussed the tools/software used to deliver the online QI training components. One study used Adobe Connect and Blackboard for delivering a web-based QI curriculum [17] while for a digital storytelling pedagogy, researchers recommended participants to use freely available software such as Windows Moviemaker or Apple iMovie [18]. Another QI collaborative used iLab which is a secure, online workspace [19] while a study that developed a web-based QI portal used WordPress CMS platform, social media account integration and a network management site called Hootsuite [22]. A microlearning app called Qstream was used in another training programme [27].

### Efficacy of QI training

Studies used various evaluation methods; some focused on programme level factors such as course completion rates [16, 27], learner reaction to training [14, 17, 25, 32], engagement level of participants [14, 26, 28], participant perceptions of the online module content and quality [20], preferred training delivery mode [16, 22, 27] and document and report submission by participating teams [32]. Studies evaluated the impact of training on participant comfort [31] and confidence [20, 28] in using QI. Many studies also assessed improvement in participants’ QI knowledge, skills, attitudes and behaviours as a result of the training [14–17, 21, 23–25, 27, 31]. Relatively few studies measured improvement in patient safety skills and knowledge of participants post-programme [23, 24]. How participants implemented QI skills and knowledge also constituted a part of outcome evaluation in various studies. This included development of action and improvement plans [18], number of QI projects developed [20] and completed [28] and results attained from these projects [19]. Improved results for the patients were also used as a proxy for outcome evaluation [14, 15, 17–19, 27, 30, 32].

Most of the included studies reported achieving desired outcomes such as improved QI knowledge and skills [14, 21, 24, 25, 27, 28], positive reaction from participants towards the training [17, 19, 20, 32], implementation of QI knowledge by participants [18, 29] and confidence to use the learned skills in future [22, 28]. One study reported no improvement in the measure being tracked [32]. In the studies with control groups, the intervention participants demonstrated improved QI knowledge [16], improved comfort with QI methods [31] and greater participation than the control groups [26]. One study did not demonstrate any significant difference in post-intervention safety indicators between intervention and comparison group [30].

This shows that majority of the interventions were successful in demonstrating the desired results. However, there was scant information around the role played by mode of delivery in the attainment of these outcomes. One study using a control group concluded that use of online content in conjunction with in-person sessions as being more effective in improving QI knowledge than only relying on online content [23]. The flipped curriculum [16] and utilisation of web-based platforms to deliver advanced QI training [17] proved to be effective methods for teaching QI. Since there is a shortage of comparable prior studies on web-based tools for QI education, it is challenging to compare results across similar interventions and more longitudinal studies may be required to analyse outcome trends over time [22].

### Benefits of online QI education

An online QI programme can virtually connect users and provide them with an environment that balances training and practice [22]. Online delivery of QI training programmes requires fewer resources [19], reduces the burden on sites and instructors [17] and the organisations do not need to maintain QI faculty [17]. It is useful in delivering a centralised QI curriculum to distributed learners [17] hence increasing the reach of the programme [19, 27]. Additionally, online learning seemed to balance the educational time constraints and clinical responsibilities in educating healthcare professionals [16]. Moreover, the IHI teaching modules used in this study are widely recognised and accessible to all programmes and learners [16]. Interactive, distance learning modules which occur in real time with multi-way communication, feedback and tailored education proved to be effective [17]. Online modules which are interactive were preferred by participants over static computer-based modules [16].

Participants in online QI programmes have more control over their learning time [27], allowing them to complete much of the curriculum at a time convenient to them [31]. Programmes that are interactive and real-time in nature lead to better and personalised engagement from participants [17]. In the case of a flipped classroom where participants complete online modules prior to in-person sessions, the online component enables maximisation of in-class time [21]. However, many participants expressed that in-class sessions and in-class application were more effective than online content in enhancing their QI knowledge [16]. Similarly, viewing didactic material on videos beforehand enables participants to use classroom time to clarify concepts [25] and reduces the overall time required for the curriculum [20]. Alternatively, quiz-based online courses provide real-time feedback, engagement and healthy competition, but are more suited to reinforce concepts taught in classrooms and supplement other QI activities rather than as standalone activities [27].

Another advantage of online programmes using innovative methods such as digital story telling is that it engages the participants in learning a new skill and creates a level playing field in terms of the anxiety associated with a new experience [18]. Virtual discussion boards also have the advantage of providing a safe space where participants can freely express their opinions and ideas which they might not feel comfortable doing face-to-face [18]. Similarly, using game mechanics and team-based competition in a safe virtual environment is an effective participant engagement strategy [26].

Participants were generally positive about features of online programmes such as open discussion forums, closed groups, private messaging, and feedback submission forms [22]. Using tools such as leader boards motivates participants to engage and provides a sense of status [26]. Group size between two to eight participants worked best [21]. Virtual formats also allow for easier modifications in the curriculum length, content, and level which are important considerations in training design [17].

Open communication, stakeholder buy-in, and continuous feedback were necessary in developing a shared vision and QI site ownership [22]. Educational content developed by faculty with practical and teaching QI experience strengthens the programme [27]. The early involvement of key stakeholders and SMART (specific, measurable, attainable, relevant, and time-bound) goals proved to be critical to the success [29]. QI coaches also play an important role in distance learning programmes as well and a study concluded that having tailored coaching support for each team was a useful aspect of the programme [14]. In the case of students, providing an opportunity for experiential learning through QI project completion alongside a faculty member was also important [31].

### Limitations of online methods

Evidence suggests that mobile and asynchronous educational technologies have the potential to overcome barriers related to teaching QI methods [26]. However, studies have also identified some limitations to such approaches. Participants often valued the learning application sessions conducted in person, more than the online components [16]. In the same way, although learners enjoyed asynchronous learning and online delivery, they preferred assessment questions that focused on application of concepts rather than information acquisition [27]. Online modes offer limited networking opportunities [19].

Customising didactic materials to suit programme and participant needs is a time-intensive task [28]. Developing an online site can be resource-intensive and lead to functional problems [19]. Since data and reporting systems are external and independent from the QI education sites, it is difficult to integrate these as site resources [22]. Online programmes also require facilitators who have QI knowledge [32] as well as technical support in case participants face any technical challenges [20] such as phone line chatter as reported in a study [32]. Additionally, the adoption and use of a new technology requires significant run-time [22]. Although participants are familiar with the use of devices such as mobile phones and computers, their use may be limited because of text character restrictions and email fatigue [27]. Although the IHI Open School modules are widely used and effective, one study recommended augmenting the content to suit local needs [23] and online content such as videos should be at an appropriate level and pace suitable to the participants [25].

With blended learning programmes, a major challenge was group session scheduling so that participants could attend without disruption of clinical responsibilities [28]. Some participating teams raised concerns specific to collecting and reporting the measurement data and perceived the measures to be complex and not well-matched to the teams’ goals [19]. Apart from the challenges associated with online delivery, teams also experience other challenges such as demand of other work duties and inability to meet as a team during implementation [14].

## Discussion

The training modalities discussed in the included studies can be broadly categorised into e-learning programmes and blended learning programmes. A previous systematic review comparing online with face-to-face education for healthcare professionals concluded that online programmes had comparable knowledge gains and benefits to onsite or face-to-face training [33]. However, there is a gap in literature evaluating the success of QI training delivery online. The purpose of this evidence review is to focus on efficacy of QI training and education interventions being delivered online to synthesise recommendations for adapting QI training content into e-learning materials.

The review highlights that distance learning approaches to QI help in overcoming barriers to traditional QI training such as shortage of trained faculty and deficiencies in organisational structure to support QI education [24]. Additionally, many participants preferred blended approaches to traditional approaches [32]. This review highlights important lessons for future programmes including balancing virtual and non-virtual methods, improving the technology and providing resources and support specific to learners [19]. Like other QI programmes, distance learning QI education also requires substantial commitment from the organisation, collaboration among participants, faculty, and leaders for success [14].

Another recommendation for those considering online delivery of QI programmes is to build relationships with institutional QI units to identify resources and link the programme to an institutional network of QI education [28]. Instead of developing home-grown solutions, future collaboratives can purchase or customise existing applications for their technical infrastructure [19]. Programmes aiming to deliver QI curriculum online also have the option of collaborating with other programmes that already have faculty with QI expertise or use publicly available online QI courses [28].

Coaches play an important role in the success of the initiatives and should connect with teams early on to provide supplemental support through coaching calls [19]. In terms of content, programme developers should also explore utilising psychological learning effects of spaced learning and testing [26] and identify more conceptually and methodologically appropriate performance measures [19]. The four levels of the Kirkpatrick Model, which measure participant reaction to the training, their learning, behavioural change and achievement of results are often used in the evaluation of training programmes [34]. An important consideration for future programme evaluation is that reactions to the training do not necessarily correspond to actual knowledge increase therefore QI curricula should be evaluated on multiple criteria rather than just on participant reaction [17]. Institutional leadership and environment play an important role in the effectiveness of QI programmes and should be considered in programme design [29].

There is a need for curriculum and training designs to evolve to the needs of the new generation of healthcare professionals with an increasing emphasis on technological tools to overcome the generational difference between educators and learners [35]. The review also highlighted that online training programmes offer better psychological safety for learners as compared to traditional programmes in medical education. This is in congruence with literature on psychological safety in medical education which postulates that medical students are better able to concentrate on their learning in a safe learning environment where there is neither a need to constantly self-monitor themselves nor a fear of being judged or scorned [36]. Some important questions to be considered in delivering QI programmes through distance learning modes are summarised in Table 3**.**

**Table 3:** Important questions for virtual training adaptation

*Capability assessment*
Do the trainees have access to the required resources and infrastructure to benefit from online delivery?
Do the trainees have the required technical understanding to participate in online training?
Does the training organisation have the necessary QI expertise and facilitation capacity?
Does the training organisation have the requisite technical support?
What are the current challenges faced by the training organisation in delivering traditional QI programmes?
*Make or buy*
Does the organisation have the required resources, skills, and technical support to develop an online QI training solution from scratch?
Does the organisation have an already available QI platform that can be adapted to deliver QI training?
Are there any already available external platforms or QI resources that the organisation can use for delivering training?
What distance learning modes does the training organisation currently incorporates in traditional QI training?
What will be the financial impact for the make vs. buy decision and does the training organisation have the required budget?
What is the opinion of the key stakeholders regarding distance learning delivery and make vs buy decision?
Does the training organisation have the resources to conduct pilot testing of the e-delivery prior to the launch?
*Structure and content*
Are the training objectives, topics, and activities suitable for distance delivery?
Does the organisation want to develop or use their own content or adapt from already available content?
Would the programme be delivered completely online, or a blended learning approach would be used?
How can the social and networking aspects of face-to-face programmes adapted to online delivery?
How will the facilitators provide distance learning support and coaching?
How will contextual factors be incorporated into programme design?
*Programme evaluation and implementation*
What is important to the training organisation in terms of evaluation? (For example: programme attendance, training reaction, patient level outcome, organisational outcomes, content knowledge testing, QI project outcomes, feedback on training mode)
How will feedback and evaluation data be collected and who will be responsible?
Will the evaluation be short term or long term?
Will the training organisation provide extended coaching online/distance coaching support and how?
What implementation support will be provided and how?

Important questions for adapting QI programme delivery to online modes

## Limitations

This rapid evidence review aimed to synthesise research and recommendations quickly and comprehensively to adapt traditional QI programmes for online delivery in response to the COVID-19 pandemic which has disrupted healthcare systems and QI training programmes. The review employed a systematic search strategy with robust and transparent screening processes. Owing to the time and resource constraints, only one reviewer was involved in the screening process. However, two additional reviewers critically reviewed the study synthesis to ensure quality. It is recognised that a systematic review is neither appropriate nor possible in every situation [37]. This rapid narrative reviews serves a specific and time-sensitive purpose and will be useful for policy-makers and QI programme designers seeking to make quick decisions about adapting training to meet needs of healthcare staff during the COVID-19 pandemic.

## Conclusion

The findings of this review have important implications for those looking to adapt traditional QI programmes to a virtual environment. Virtual training environment holds great potential in delivering standardised training remotely, which will be of utmost importance in the post-COVID environment. However, caution should be practised, and a realistic evaluation of capabilities and needs should be conducted before making adaptation decisions. Factors such as programme design, mode of delivery, technical skills, implementation support and contextual factors are important considerations. Virtual QI and training of healthcare professionals and students is a viable, efficient, and effective alternative to traditional QI education that will play a vital role in building their competence and confidence to improve the healthcare system in post-COVID environment.

## Supplementary information

**Additional file 1: **Search strategy.

**Additional file 2:** Quality assessement of included studies.

## Data Availability

The studies analysed as part of the narrative literature review are all referenced in the manuscript. No additional data and material are included.

## Abbreviations

COVID-19: Coronavirus disease 2019
QI: Quality improvement
QI-MQCS: Quality improvement minimum quality criteria set
QII: Quality improvement interventions
