# Supplementary material for: Virtual adaptation of traditional healthcare quality improvement training in response to COVID-19: a rapid narrative review
Source: Hum Resour Health. 2020 Oct 28;18:81. doi: 10.1186/s12960-020-00527-2 (PMC7594275; doi:10.1186/s12960-020-00527-2)
Supplement: Supplementary file 1 — Additional file 1: Search strategy. [file 12960_2020_527_MOESM1_ESM.docx]

**Additional File 1: Search Strategy**

**Search date:** 29^th^ May 2020

**Search duration:** 01-01-2015 till 29-05-2020

| **Database** | **Search String** | **Results** |
| --- | --- | --- |
| **PubMed** | (“Quality Improvement”[Title/Abstract] OR QI)[Title/Abstract] AND (training[Title/Abstract] OR course[Title/Abstract] OR module[Title/Abstract] OR education[Title/Abstract] OR “continuing professional development”[Title/Abstract] OR CPD)[Title/Abstract] AND (remote[Title/Abstract] OR Online[Title/Abstract] OR distance[Title/Abstract] OR virtual[Title/Abstract] OR “e-learning”)[Title/Abstract] AND Health*[Title/Abstract] | **177** |
| **Web of Science** | TS=(“Quality Improvement” OR QI) AND TS=(training OR course OR module OR education OR “continuing professional development” OR CPD) AND TS=(remote OR Online OR distance OR virtual OR “e-learning”) AND TS=Health* | **195** |
| **Scopus** | TITLE-ABS-KEY ( ( "Quality Improvement" OR qi ) AND ( training OR course OR module OR education OR "continuing professional development" OR CPD ) AND ( remote OR online OR distance OR virtual OR "e-learning" ) AND health* ) | **419** |
| **Total** | | **791** |
